# A meta-analysis of unicompartmental knee arthroplasty revised to total knee arthroplasty versus primary total knee arthroplasty

**DOI:** 10.1186/s13018-018-0859-1

**Published:** 2018-06-22

**Authors:** Xuedong Sun, Zheng Su

**Affiliations:** 1 Department of Orthopaedics, Weifang People’s Hospital, no. 151 Guangwen Road, Weifang, 260041 China; 2 Department of Medical Oncology, Weifang People’s Hospital, no. 151 Guangwen Road, Weifang, 260041 China

**Keywords:** Knee osteoarthritis, Unicompartmental knee arthroplasty, Total knee arthroplasty, Meta-analysis

## Abstract

**Background:**

This study was performed to compare the clinical outcomes of unicompartmental knee arthroplasty (UKA) revised to total knee arthroplasty (TKA) versus primary TKA.

**Methods:**

Relevant trials were identified via a search of the Cochrane Central Register of Controlled Trials and PubMed from inception to 17 June 2017. A meta-analysis was performed to compare postoperative outcomes between revised UKA and primary TKA with respect to the Western Ontario and McMaster Universities Osteoarthritis Index (WOMAC) score, Knee Society Score (KSS), mean polyethylene thickness, hospital stay, revision rate, range of motion (ROM), and complications.

**Results:**

Five of 233 studies involving 536 adult patients (revised UKA group, *n* = 209; primary TKA group, *n* = 327) were eligible for inclusion in the meta-analysis. The primary TKA group had better WOMAC scores, KSS, and ROM than the revised UKA group (*P* < 0.05). Compared with primary TKA, revision of UKA to TKA required more augments, stems, and bone grafts and a thicker polyethylene component (*P* < 0.05). There were no significant differences between the two groups in the revision rate, hospital stay, or complications (*P* > 0.05).

**Conclusion:**

Conversion of UKA to TKA is associated with poorer clinical outcomes than primary TKA. Furthermore, we believe that conversion of UKA to TKA is more complicated than performing primary TKA. Revision UKA often requires more augments, stems, and bone grafts and thicker polyethylene components than primary TKA. However, patients who undergo conversion of UKA to TKA have similar hospital stay, complications, and revision rate as patients who undergo primary TKA.

## Background

The best treatment options for patients with unicompartmental osteoarthritis of the knee are still controversial [1]. Total knee arthroplasty (TKA) and unicompartmental knee arthroplasty (UKA) are both used to treat osteoarthritis of the knee. Because of the continuous development of surgical techniques and component design since the early 1970s [2, 3], UKA has become a more successful and reliable treatment method for unicompartmental knee osteoarthritis. When UKA failure occurs, TKA is an alternative treatment for many patients. However, some authors have reported poor outcomes of conversion of UKA to TKA [4–6], whereas others have reported more favorable outcomes [7, 8]. Hence, it is important for patients to understand the potential clinical outcomes of revision surgery during their preoperative deliberation. No previous meta-analysis has compared the clinical outcomes of revised UKA versus primary TKA. Therefore, we performed a meta-analysis of clinical studies to compare revised UKA and primary TKA by evaluating knee pain, knee function, and other parameters.

## Methods

### Search strategy

The Cochrane Central Register of Controlled Trials and PubMed databases were searched to identify relevant studies published in English from inception to 17 June 2017. The following search strategy was used to maximize search specificity and sensitivity: [(revision uka) OR (revised uka) OR (revised unicompartmental knee) OR (revision unicompartmental knee) OR (revised ukr) OR (revision ukr)] AND [(total knee) OR tka OR tkr], where “ukr” stands for unicompartmental knee replacement and “tkr” stands for total knee replacement.

### Selection of studies

Two independent authors (X.D.S. and Z.S.) initially selected studies based on their titles and abstracts. Full papers were retrieved if a decision could not be made from the abstracts. Any disagreement between the two authors was resolved by consensus.

The inclusion criteria were

- Comparison of clinical outcomes between revised UKA and primary TKA
- Prospective study or retrospective study
- Cohort study, case control study, or randomized controlled trial
- Mean follow-up duration of at least 2 years
- Comparison of at least one of the following outcomes: Western Ontario and McMaster Universities Osteoarthritis Index (WOMAC) score, Knee Society Score (KSS), mean polyethylene thickness, hospital stay, range of motion (ROM), postoperative complications (nerve injury, hematoma, deep vein thrombosis, patellar tendon disruption, fractures, infection, component loosening, stiffness), and revision rates
- Sufficient data for extraction and pooling (i.e., reporting of the mean, standard deviation, and number of subjects for continuous outcomes and the number of subjects for dichotomous outcomes)

The exclusion criteria were

- Revision of infectious loosening after UKA
- Review articles or case reports
- Revision of patellofemoral replacement
- Performance of bilateral TKA or UKA

### Data extraction

Two reviewers (X.D.S. and Z.S.) independently performed data extraction using standardized data extraction forms. The general characteristics of each study were extracted [i.e., mean age, sex, body mass index (BMI), ROM, mean polyethylene thickness, hospital stay, postoperative complications, revision rate, KSS, and WOMAC score]. Any disagreement between the two reviewers was resolved by consensus.

### Quality assessment

Both authors (X.D.S. and Z.S.) independently assessed the risk of bias for each study in accordance with the Newcastle–Ottawa scale (Table 1). Three domains were assessed, and the total possible score was 9 points. Disagreements between the two authors were resolved by consensus.

**Table 1:** Newcastle–Ottawa scale

Study	Selection				Comparability		Exposure			Quality score
	Cases definition	Cases representativeness	Controls selection	Controls definition	Comparable for a, b, c*	Comparable for d, e, f*	Exposure ascertainment	Controls ascertainment	Non-response rate	
Järvenpää J [5]	1	0	0	1	a, b, c	d, f	1	1	1	7
Rancourt MF [9]	1	0	0	1	a, b, c	f	1	1	1	7
Becker R [10]	1	0	0	1	a, b, c	f	1	1	1	7
^Lunebourg^ A [11]	1	0	0	1	a, b, c	e	1	1	1	7
Cross MB [12]	1	0	0	1	a, b, c	NA	1	1	1	6

*NA* data not available

Comparability ^variables^: *a* = age; *b* = sex; *c* = body mass index; *d* = operation time point; *e* = single surgeon; *f* = the same compartment

*If all characteristics of *a*, *b*, and *c* were comparable, 1 point was assigned; if one, two, or three characteristics of *d*, *e*, and *f* were comparable, 1 point was assigned; otherwise, 0 points were assigned

### Statistical analysis

Dichotomous outcomes are expressed as the risk ratio (RR) with 95% confidence interval (CI), while continuous outcomes are expressed as the mean difference (MD) with 95% CI. Heterogeneity is expressed as *P* and *I*^2^. This value of *I*^2^ ranges from 0% (complete consistency) to 100% (complete inconsistency). If the *P* value of the heterogeneity test was < 0.1 or *I*^2^ > 50%, a random-effects model was used in place of the fixed modality. Publication bias was tested using funnel plots. Forest plots were used to graphically present the results of individual studies and the respective pooled estimate of effect size. All statistical analyses were performed with Review Manager (version 5.3.0 for Windows; Cochrane Collaboration, Nordic Cochrane Centre, Copenhagen, Denmark).

## Results

### Search results

**Fig. 1:**
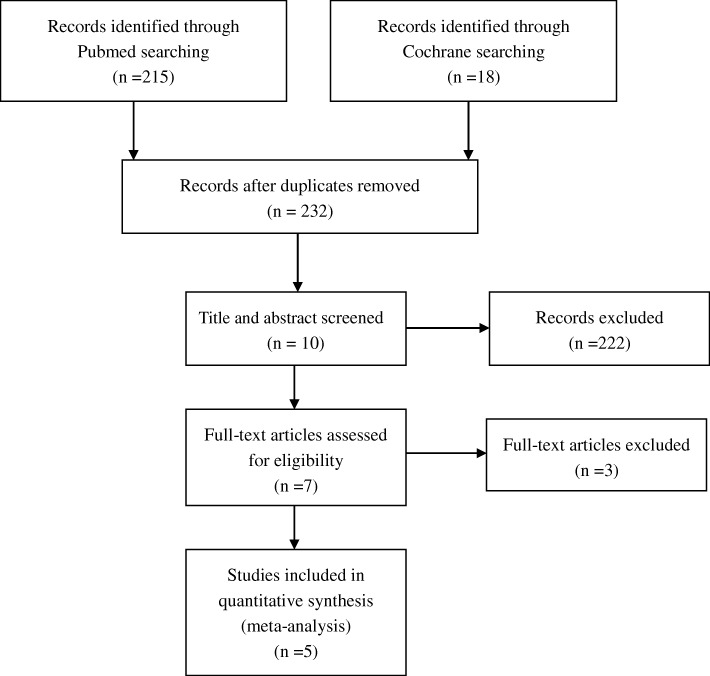
Flowchart of study selection

A flowchart of the studies considered for inclusion in our review is shown in Fig. 1. We identified 233 potential citations (215 from PubMed, 18 from the Cochrane Library) comparing the clinical outcomes of revised UKA and primary TKA. After reading the articles, 5 of the 233 citations were selected for the meta-analysis. The characteristics of these five studies [5, 9–12] are shown in Table 2.

**Table 2:** Characteristics of included studies

References	Years	Patients (*n*) rUKA/pTKA	Mean age (years) rUKA/pTKA	Female rUKA/pTKA	Mean follow-up (years)	Mean BMI (kg/m^2^) rUKA/pTKA	Outcome
Järvenpää J [5]	2010	21/28	74.9(7.4)/75.2(7.2)	12/17	10.5	28.5(4)/30.5(4.4)	Hospital stay, ROM, WOMAC scores, revisions, complications, requirement of augments, stems, and bone grafts
Rancourt MF [9]	2012	63/126	67.49(10.24)/66.71(9.77)	45/90	3	31.6(6.15)/32.53(6.57)	Hospital stay, WOMAC scores, mean polyethylene thickness, requirement of augments, stems, and bone grafts
Lunebourg A [11]	2015	48/48	71(9)/72(12)	36/32	7	28(4)/28(4)	ROM, KSS, mean polyethylene thickness, revisions, complications, requirement of augments, stems, and bone grafts
Becker R [10]	2004	28/28	71.5(6.8)/71.5(6.6)	23/23	4.6	31.2(3.2)/31.1(4.4)	ROM, WOMAC scores, KSS
Cross MB [12]	2014	49/97	61.5/58.9	30/50	4.8	31.65/32.76	hospital stay, ROM, KSS, revisions, complications, requirement of augments, stems, and bone grafts

*rUKA* revised unicompartmental knee arthroplasty, *pTKA* primary total knee arthroplasty, *BMI* body mass index, *ROM* range of motion, *WOMAC* Western Ontario and McMaster Universities Osteoarthritis Index, *KSS* Knee Society Score

### Meta-analysis results

**Fig. 2:**
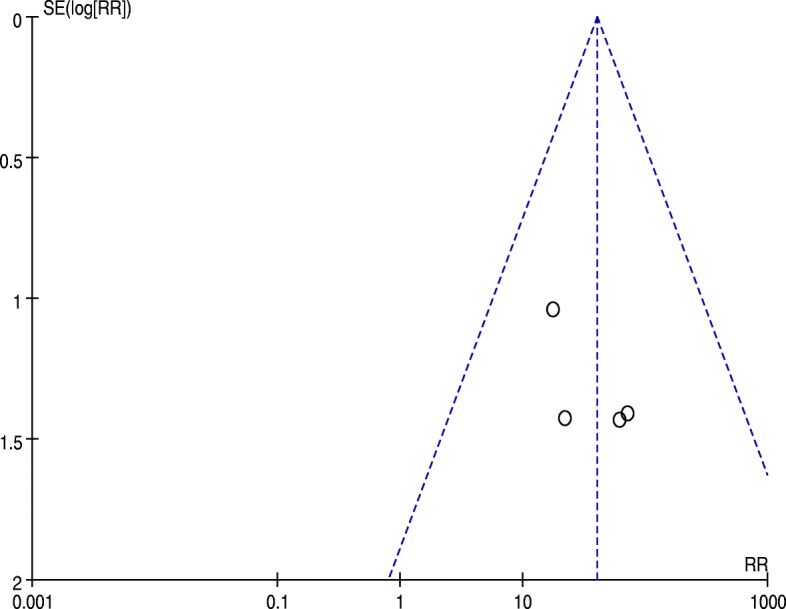
Funnel plot for requirement of augments, stems, and bone grafts

The meta-analysis included five studies, involving a total of 536 patients [5, 9–12]. The revised UKA group included 209 patients, while the primary TKA group included 327 patients. The MD for age and BMI were 0.43 (*P* = 0.61; 95% CI, − 1.24–2.10) and − 0.67 (*P* = 0.13; 95% CI, − 1.56–0.21), respectively; there were no significant differences between groups in age or BMI. There was also no significant difference between groups in the proportion of female patients (RR = 1.06; *P* = 0.36; 95% CI, 0.94–1.19). Thus, the age, sex, and BMI of the two groups were comparable. A funnel plot based on the most frequently cited outcome was broadly symmetrical, indicating minimal publication bias (Fig. 2).

#### Hospital stay, complications, and revision rates

**Fig. 3:**
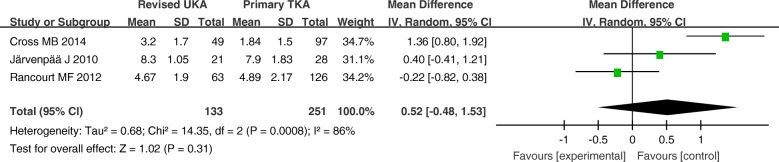
Forest plot for hospital stay

**Fig. 4:**
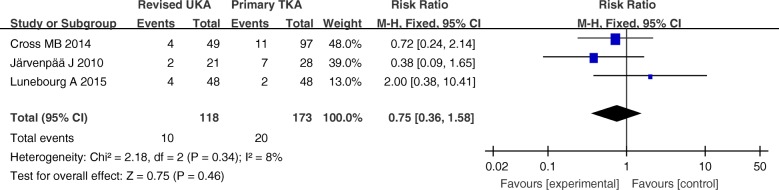
Forest plot for complications

**Fig. 5:**
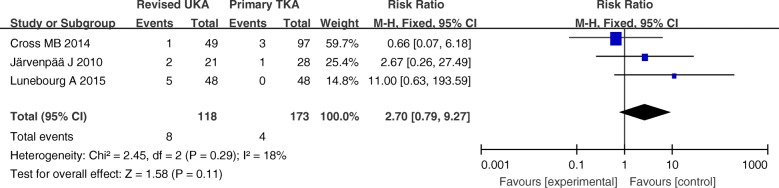
Forest plot for revision rates

The hospital stay, complications, and revision rates are summarized in Figs. 3, 4, and 5. There were no significant differences between these variables in the primary TKA group versus the revised UKA group (*P* > 0.05).

#### WOMAC scores, KSS, and ROM

The WOMAC score (0–100) encompasses evaluation of the knee as well as patients’ symptoms and functional disability. The score has three main categories: pain, stiffness, and function. The KSS consists of the Knee Society Knee Score (KKS 0–100) and the Knee Society Function Score (KFS 0–100).

**Fig. 6:**
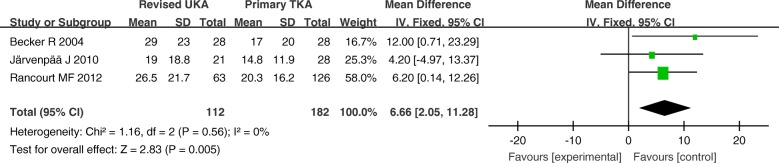
Forest plot for Western Ontario and McMaster Universities Osteoarthritis Index (WOMAC) function scores

**Fig. 7:**
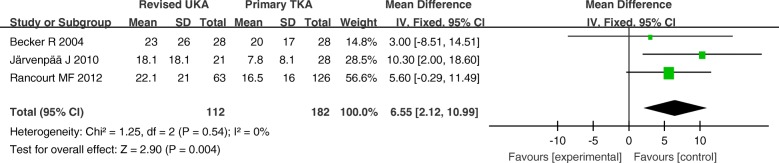
Forest plot for Western Ontario and McMaster Universities Osteoarthritis Index (WOMAC) pain scores

**Fig. 8:**
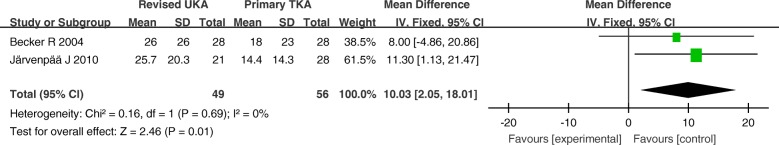
Western Ontario and McMaster Universities Osteoarthritis Index (WOMAC) stiffness scores

The MD of the WOMAC function, pain, and stiffness scores (0–100) for revised UKA were 6.66 (*P* = 0.005; 95% CI, 2.05–11.28), 6.55 (*P* = 0.004; 95% CI, 2.12–10.99), and 10.03 (*P* = 0.01; 95% CI, 2.05–18.01), respectively, all of which were higher than those for primary TKA. The WOMAC scores were significantly different between the two groups (Figs. 6, 7, and 8).

**Fig. 9:**
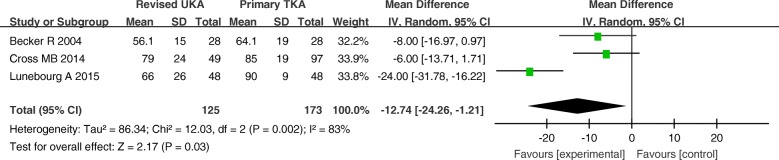
Forest plot for Knee Society Function Score

The MD of the KFS for revised UKA was − 12.74 (*P* = 0.03; 95% CI, − 24.26 to − 1.21), which was lower than that for primary TKA. There was a significant difference in the KFS was observed between the two groups (Fig. 9).

**Fig. 10:**
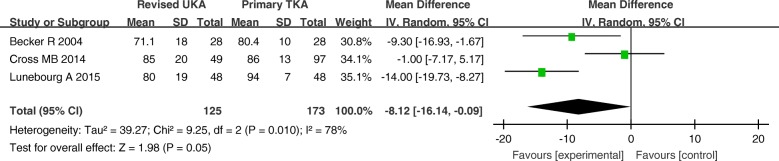
Forest plot for Knee Society Knee Score

**Fig. 11:**
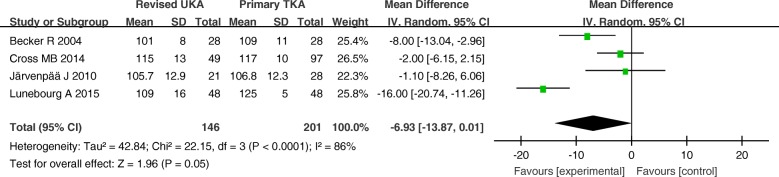
Forest plot for range of motion (ROM)

The MD of the KKS and ROM for revised UKA were − 8.12 (*P* = 0.05; 95% CI, − 16.14 to − 0.09) and − 6.93 (*P* = 0.05; 95% CI, − 13.87–0.01), respectively. These results imply that the ROM and KKS tended to be better in the primary TKA group than that in revised UKA group, but the differences between the two groups were not statistically significant (Figs. 10 and 11).

#### Polyethylene thickness and requirement for augments, stems, and bone grafts

**Fig. 12:**
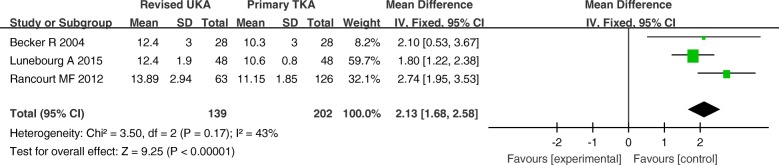
Forest plot for polyethylene thickness

Three studies involving 341 patients provided data on polyethylene thickness. The polyethylene thickness used for the revised UKA group was significantly thicker than that used for the primary TKA group (MD = 2.13; 95% CI, 1.68–2.58; *P* < 0.00001) (Fig. 12).

**Fig. 13:**
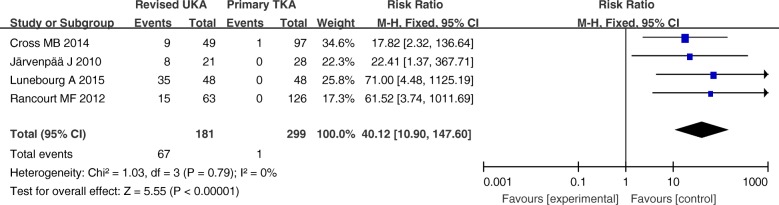
Forest plot for requirement of augments, stems, and bone grafts

Four studies involving 480 patients provided data on the requirements for augments, stems, and bone grafts. There was a significantly greater proportion of usage of augments, stems, and bone grafts in the revised UKA group than in the primary TKA group (RR = 40.12; *P* < 0.00001; 95% CI 10.90–147.60) (Fig. 13).

## Discussion

The most important finding of the present meta-analysis was that the primary TKA group showed better outcomes than the revised UKA group in terms of WOMAC scores, KSS, and ROM. There was a greater proportion of usage of augments, stems, and bone grafts in the revised UKA group than in the primary TKA group, and the polyethylene thickness used for the revised UKA group was thicker than that used for the primary TKA group. However, there were no significant differences between the revised UKA group and the primary TKA group in the hospital stay, complications, and revision rates.

In our review, the primary TKA group yielded superior KSS and WOMAC pain, stiffness, and function scores compared with the revised UKA group. Lunebourg et al [11] and Miller et al [6] reported that the mean KSS was significantly worse in the revised UKA group than that in the primary TKA group, whereas Cross et al [12] did not favor this view. Järvenpää et al [5] stated that the outcomes of WOMAC pain and stiffness scores were better in the primary TKA group, and the WOMAC function scores did not significantly differ between the revised UKA group and the primary TKA group; however, Becker et al [10] reported the opposite. These studies only used a single questionnaire to evaluate each patient. The KSS only evaluates walking and stair-climbing activities, whereas the self-assessed WOMAC scores assess the ability of the patient to perform activities of daily living in more detail. Therefore, the results of the two groups were able to be evaluated more comprehensively with the combination of objective and subjective outcome systems used in our study.

ROM is one of the most important clinical outcomes that reflects the function of the knee. The revised UKA group had decreased ROM compared with the primary TKA group in the present study, which is in accordance with other studies [10–12]. Scarring or thickening of the joint capsule is more likely after revision surgery, and this may be partially responsible for the decreased knee flexion. Therefore, early recognition and enhanced recovery after surgery are critical for successful outcomes.

Bone loss is reportedly experienced by 77% of patients who undergo conversion of UKA [8]. Bone defects reportedly occur in 60.6% of the cases [13], and bone loss can also occur at the time of component removal [14]. Some studies have also verified this view from other aspects; 34% of patients required conversion to a revision type of TKA with augments, stems, or bone grafts [15], and 33% of cases reportedly require revision components (with the majority on the tibial side) [16]. Furthermore, UKA to TKA conversion was often accompanied by the use of thicker polyethylene [9, 10, 17]. Wynn Jones et al. [18] reported that UKA to TKA conversion with a thicker polyethylene was related to the initial polyethylene thickness of the UKA, and that these cases with thicker polyethylene more often needed an augment or a stem. In the present meta-analysis, we found a greater proportion of usage of augments, stems, and bone grafts and a thicker polyethylene component in the revised UKA group than in the primary TKA group; this indicates that the revised operations were more complicated, and thus required excellent surgical technique. Therefore, we believe that converting UKA to TKA is more difficult than performing primary TKA. In UKA revision, surgeons should perform adequate preoperative preparation to ensure successful operation.

UKA is still a successful and reliable treatment method for unicompartmental knee osteoarthritis. Previous studies have revealed that UKA results in less perioperative blood loss, a shorter hospital stay, fewer complications, better ROM, greater level of activity, more normal gait, and a subsequently quicker recovery compared with TKA [19–21]. Moreover, one retrospective series of patients undergoing UKA reported an 11-year survival rate of 92% [22], and another study reported a 12-year survival rate of 94% among patients aged ≤ 60 years [23]. However, with the widespread use of UKA, a greater early revision rate of UKA has been reported. Two previous studies reported that patients undergoing UKA were at greater risk of early revision than those undergoing primary TKA [24, 25]; however, these studies did not account for surgeon proficiency. Surgeon experience is essential for the attainment of good results in UKA [26]. The reported revision rates for UKA are 0.99% for UKA conducted by surgeons performing > 12 UKAs per year, 4.6% for those performing 8 to 11 UKAs per year, 6.4% for those performing 2 to 7 UKAs per year, and 8.3% for those performing 1 UKA per year [27]. In addition, a study evaluating the published long-term outcomes of > 8000 medial Oxford Phase 3 UKAs reported that very good outcomes were achieved by both designer and non-designer surgeons, and that the annual revision rate was 0.74% [28]. In conclusion, UKA has a greater long-term survival rate because of improved surgical techniques and modern implant designs along with increased experience with the procedure. Therefore, higher-volume surgeons can achieve better UKA outcomes and a revision rate comparable with that of TKA, but TKA may be a wiser choice for less experienced surgeons.

The strengths of the study are the compatibility of the patient populations in terms of age, sex, and BMI, and the use of both objective and subjective data. The limitations include the insufficient sample size, different types of prostheses used, and lack of survival rate calculation. Future studies with large sample sizes could provide enhanced analyses, and additional evaluation criteria are needed.

## Conclusion

The present meta-analysis has shown that conversion of UKA to TKA is associated with poorer clinical outcomes than primary TKA. Furthermore, we believe that converting UKA to TKA is more complicated than performing primary TKA. Surgeons should be aware that revision UKA more often requires augments, stems, and bone grafts and thicker polyethylene components than primary TKA. However, there are no statistically significant differences between the two groups in the hospital stay, complications, or revision rates.

## Abbreviations

BMI: Body mass index
CI: Confidence interval
KFS: Knee Society Function Score
KKS: Knee Society Knee Score
KSS: Knee Society Score
MD: Mean difference
ROM: Range of motion
RR: Risk ratio
TKA: Total knee arthroplasty
UKA: Unicompartmental knee arthroplasty
WOMAC: Western Ontario and McMaster Universities Osteoarthritis Index
